# Particulate Matter Exposure and Attention-Deficit/Hyperactivity Disorder in Children: A Systematic Review of Epidemiological Studies

**DOI:** 10.3390/ijerph17010067

**Published:** 2019-12-20

**Authors:** Gabriele Donzelli, Agustin Llopis-Gonzalez, Agustin Llopis-Morales, Lorenzo Cioni, María Morales-Suárez-Varela

**Affiliations:** 1 Department of Preventive Medicine and Public Health, Food Sciences, Toxicology, and Legal Medicine, School of Pharmacy, University of Valencia, Avenida Vicente Andres Estellés s/n, Burjassot, 46100 Valencia, Spain; agustin.llopis@uv.es (A.L.-G.); agustinllopis@gmail.com (A.L.-M.); Maria.M.Morales@uv.es (M.M.-S.-V.); 2 Biomedical Research Consortium in Epidemiology and Public Health Network (CIBERESP), Avenida Monforte de Lemos, 3-5, Pabellón 11, Planta 0 28029 Madrid, Spain; 3 Scuola Normale Superiore, Piazza dei Cavalieri, 7-56126 Pisa, Italy; lorenzo.cioni@sns.it

**Keywords:** air pollution exposure, particulate matter (PM), environmental epidemiology, environmental pollution, attention-deficit/hyperactivity disorder (ADHD), public health policy

## Abstract

Attention-deficit/hyperactivity disorder (ADHD) is the most common cognitive and behavioural disorder affecting children, with a worldwide-pooled prevalence of around 5%. Exposure to particulate matter (PM) air pollution is suspected to be associated with autism spectrum disorders and recent studies have investigated the relationship between PM exposure and ADHD. In the absence of any synthesis of the relevant literature on this topic, this systematic review of epidemiological studies aimed to investigate the relationship between the exposure of children to PM and ADHD and identify gaps in our current knowledge. In December 2018, we searched the PubMed and EMBASE databases. We only included epidemiological studies carried out on children without any age limit, measuring PM exposure and health outcomes related to ADHD. We assessed the quality of the articles and the risk of bias for each included article using the Newcastle–Ottawa Scale and the Office of Health Assessment and Translation (OHAT) approach, respectively. The keyword search yielded 774 results. Twelve studies with a total number of 181,144 children met our inclusion criteria, of which 10 were prospective cohort studies and 2 were cross-sectional studies. We subsequently classified the selected articles as high or good quality studies. A total of 9 out of the 12 studies reported a positive association between PM exposure to outdoor air pollution and behavioral problems related to attention. Despite these results, we found a significant degree of heterogeneity among the study designs. Furthermore, 11 studies were judged to be at a probably high risk of bias in the exposure assessment. In conclusion, we opine that further high quality studies are still needed in order to clarify the association between PM exposure and ADHD diagnosis.

## 1. Introduction

Diagnostic and Statistical Manual of Mental Disorders 5, Fifth Edition (DSM-5) defines attention-deficit/hyperactivity disorder (ADHD) as a persistent pattern of inattention and/or hyperactivity-impulsivity that interferes with functioning or development. To date, ADHD is the most common cognitive and behavioural disorder affecting children, with a worldwide-pooled prevalence of around 5% [1]. Recent studies have shown that in a substantial number of cases, ADHD does not remit in puberty but persists into adolescence and adulthood [2]. However, from our examination of the literature on these topics, we found no studies comparing the persistence of ADHD in adults with exposure to air pollution.

The causes of ADHD have not yet been completely understood [3]. Twin and adoption studies have shown that heritability of ADHD is between 60% and 90% [4]. However, it remains unclear how the genes interact with each other and how the interactions between environmental and genetic factors unfold.

The identification of preventable risk factors, such as environmental pollution exposure, must be given the highest priority since ADHD may have a profound impact on the children affected and their families [5].

Epidemiology has contributed to a better understanding of the relationship between environmental risk factors and ADHD. In fact, numerous epidemiological studies have focused on the role that environmental pollution exposure plays in the development of ADHD. For example, pre or postnatal lead exposure in children was associated with ADHD symptoms [6,7] as well as polycyclic aromatic hydrocarbons [8] and second-hand smoke exposure [9].

Outdoor air pollution and particulate matter (PM), one of its major components, is one of the most serious environmental risks [10]. An etiologic role of air pollution exposure on neurodevelopmental disorders is biologically plausible, although the detailed mechanisms remain elusive [11]. PM is a mixture of solid particles and liquid droplets found in the air that can be inhaled and can cause serious health problems [12]. Exposure to PM air pollution is suspected to be associated with autism spectrum disorders [13], and recent studies have investigated the possible association between PM exposure and ADHD [14,15].

In the absence of any synthesis of the relevant literature on these topics, this systematic review of epidemiological studies aimed to investigate the relationship between childhood exposure to PM and ADHD and identify some gaps in our current knowledge.

## 2. Methods

This systematic review was developed according to the Preferred Reporting Items for Systematic Reviews and Meta-Analyses (PRISMA) statement [16]. The complete PRISMA checklist is located in File S1.

### 2.1. Search Strategy

A systematic literature search was conducted in two different electronic databases: EMBASE and MEDLINE (accessed from PubMed). We only included epidemiological studies carried out on children without any age limit, measuring PM exposure and health outcomes related to ADHD. Therefore, we discarded both neurophysiological and neuropsychological studies as well as purely psychological or psychiatric studies to focus our attention on papers that emphasized the interrelations between children and their environment.

To conduct the literature searches, we used a combination of Medical Subject Headings (MeSH) and non-MeSH keywords related to PM as the exposure of interest and ADHD as the outcome. Specifically, we used the keywords PM, particulate matter, pollut *, ADHD, attention deficit, and hyperactivity disorder in the following query:

(PM OR particulate matter OR pollut *) AND (“ADHD” OR “attention deficit” OR “hyperactivity disorder”).

### 2.2. Study Selection

After removing duplicates, we evaluated titles and abstracts according to the study selection criteria exhibited in File S2 by two independent reviewers (L.C. and F.A.). Only epidemiological studies that were written in English and investigated the relationship between PM exposure and ADHD were included, whereas reviews, letters to the editor, abstracts, controlled trials, case reports, interventional studies, and in vitro and animal studies were not. In the case of inconsistency between reviewers, the third reviewer (G.D.) assessed the eligibility of the study to be included in our review.

### 2.3. Data Extraction

All relevant data were extracted, including author(s) name(s), publication date, title, location of study, study design, age group(s) of participants, sex distribution of participants, sample size, exposure assessment methods, outcome characterization approaches, statistical analysis approaches, point estimate and confidence intervals of crude and adjusted effect size(s), and level of adjustment.

### 2.4. Assessment of the Studies

#### 2.4.1. Quality Assessment

We used the Newcastle–Ottawa scale (NOS) [17] to evaluate the quality of the cohort studies. The NOS contains eight items grouped into three dimensions, including election, comparability, and outcome. A maximum of one star for each numbered item within the selection and outcome categories can be awarded. On the other hand, a maximum of 2 stars can be awarded to the comparability category. The maximum score that can be assigned in each study is therefore 9. The total score gives an indication of the overall quality of a study: 8–9 stars indicate very good studies, 6–7 good studies, 4–5 satisfactory studies, and 0–3 unsatisfactory studies (see File S3). In addition, we used a modified version of NOS. This scale was adapted from the original Newcastle–Ottawa instrument in order to assess the quality of the cross-sectional studies [18]. This assessment contains 7 items grouped into the same three dimensions. A maximum of 1 star can be assigned to each item, except for two items that can be scored from 0 to 2 stars. The maximum score and the interpretation of the total score are the same as those used in the NOS for the cohort studies (see File S3).

#### 2.4.2. Risk of Bias Assessment

Assessment of risk of bias was performed using the adapted Office of Health Assessment and Translation (OHAT) approach by the National Institutes of Environmental Health Sciences National Toxicology Program [19] and Navigation Guide by the University of California [20,21,22]. Risk of bias assessment was conducted for each included study according to key criteria (exposure assessment, outcome assessment, confounding bias) and other criteria (selection bias, attrition/exclusion bias, selective reporting bias, conflict of interest, other sources of bias). The risk of bias rating was developed, answering a set of questions based on the details of the studies in order to obtain one of the following judgments: “Low”, “probably low”, “probably high”, or “high” risk (see File S3).

## 3. Results

### 3.1. Search Results and Study Characteristics

**Figure 1:**
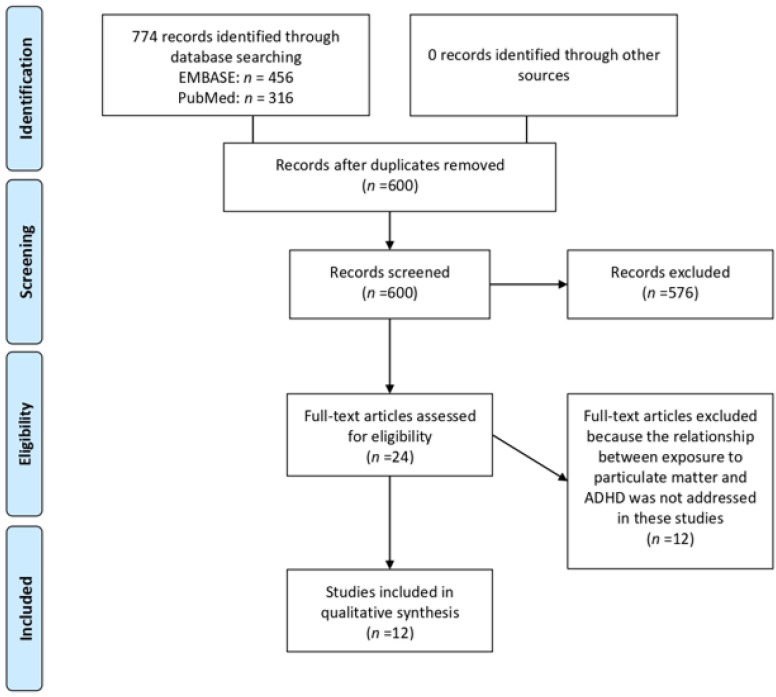
Preferred Reporting Items for Systematic Reviews and Meta-Analyses (PRISMA) flow diagram.

The article selection process for the inclusion of studies in the present review is illustrated in the PRISMA flow diagram in Figure 1. We identified 774 articles by searching only relevant databases and not other sources. From these records, 600 remained after removing the duplicates. We reduced the number of included studies to 24 after screening the titles and abstracts. These 24 articles underwent a full-text evaluation, which brought the total number down to 12 published articles that met our inclusion criteria. The main characteristics of the studies included in this review are summarized in Table 1 in order by date of publication. Among the 12 studies, there were 10 cohort ([14,15,23,24,25,26,27,28,29,30]) and two cross-sectional studies ([31,32]). Table 2 shows the results of the eight articles with reported odds ratios. Forest plot graphs give a visual suggestion of the heterogeneity of the associations.

#### 3.1.1. Prospective Cohort Studies

Our review identified eight prospective cohort studies, with a total sample size of 181,144. The sample size across these studies varied from 2618 [27] up to 66,283 [14]. We included samples drawn from 10 different countries in these studies: Denmark, the Netherlands, Germany, France, Italy, Spain, Japan, Korea, Sweden, and the US (Boston). The children’s ages ranged from 3 years to 14 years. Forns et al., 2018 [15] assessed whether NO_2_ and PM exposure during pregnancy is associated with an increased risk of ADHD symptoms in 29,127 children between 3 and 10 years old from eight European population-based birth cohorts. In this research, no evidence was found to link air pollution and ADHD symptoms. Markevych et al., 2018 [14] enrolled 66,823 children who were born between 2000 and 2004 and were residing in Saxony. The authors found that an increase of PM_10_ exposure raised the relative risk of ADHD diagnosis. Yorifuji et al., 2017 [24] examined the effect of prenatal exposure to outdoor air pollution on child behavioural problems. A total of 39,911 children of school age were enrolled in the study, and prenatal PM exposure to outdoor air pollution was associated with behavioural problems related to attention. Min et al., 2017 [26] also provide evidence of the association between PM_10_ exposure and the incidence of childhood ADHD. Yorifuji et al., 2016 [23] examined associations between prenatal exposure to PM_7_ and child behavioural development related to attention. The study showed that traffic-related air pollution exposure was positively associated with the risk of behavioral developmental delays. Basagaña et al., 2016 [27] enrolled 2618 schoolchildren (average age, 8.5 years) belonging to 39 schools in Barcelona. High levels of traffic-related PM_2.5_ showed a slower cognitive development of schoolchildren. Chiu et al., 2016 [28] evaluated the association between prenatal particulate matter with a diameter ≤2.5 μm (PM_2.5_) and children’s attention and response inhibition. This last paper showed a non-significant association between air pollution exposure and attention domains. Fuertes et al., 2016 [29] reported that PM exposure was positively associated with hyperactivity/inattention in 15-year-old German adolescents. Finally, Gong et al., 2014 [30] did not find an association between prenatal or postnatal exposure to traffic-related air pollution and neurodevelopmental disorders in children.

#### 3.1.2. Cross-Sectional Studies

Our review identified two cross-sectional studies with a total sample size of 2129. Saenen et al., 2016 [31] thoroughly investigated whether the neurobehavioral performance was different from acute versus chronic air pollution exposure in a panel of 310 primary school children in Belgium (grades 3 to 6). The results showed that PM_2.5_ and PM_10_ exposures were significantly associated with selective attention. Siddique et al., 2010 [32] explored whether sustained exposure to vehicular air pollution affects the behaviour and activities of children (aged 9–17 years) in India. The results showed a possible association between PM_10_ exposures and ADHD.

### 3.2. Assessment of Studies

#### 3.2.1. Quality Assessment

The selected cohort and cross-sectional studies received a minimum 7-star rating on the Newcastle–Ottawa scale (Table 3). Based on these results, all manuscripts included in this systematic review can be considered at a minimum good quality studies. The readers can find more information about each quality study assessment in File S3.

#### 3.2.2. Risk of Bias Assessment

The heat map illustrating this rating process is provided in Table 4. It is important to note that the risk of bias of outcome assessment varies for the different studies included in this systematic review. The articles with a diagnosis based on a psychiatric assessment were classified as having a low risk of bias and should be considered to be of higher quality. The readers can find more information about each risk of bias study assessment in File S3. None of the 12 articles were excluded for being assessed at a high risk of bias, and the majority of them were rated with “low risk” and “low probability risk” in most domains. However, some domains were rated as “high probability risk” within the three key criteria, especially for exposure assessment, which is considered one of the most critical components in these types of epidemiological studies [33].

## 4. Discussion

To the best of our knowledge, the present study provides the first systematic review to widely explore the literature of epidemiological studies investigating the possible association between environmental exposure to PM and ADHD in children. We performed a broad literature search, and found a total of 12 epidemiological studies that met the selection criteria. During this review, we found high heterogeneity in terms of study designs, sample sizes, outcomes, exposure assessment methods, and qualities. The effect of traffic-related air pollution on cognitive, behaviour, and psychomotor disorders appears to be biologically plausible. Evidence accumulated from human epidemiological and animal studies indicates that ambient air pollution may be associated with diseases of the central nervous system (CNS) [34]. Children are particularly susceptible to air pollutants, and pre- and/or postnatal exposure may negatively affect their CNS [35]. Recently, several investigation studies have integrated magnetic resonance imaging of the brain with epidemiology, showing that long-term exposure to air pollution might have adverse impacts on the human brain [36]. A recent study has shown that prenatal exposure to PM_2.5_ may be associated with a corpus callosum (CC) volume decrease in children. This air pollution-related volume decrease may be associated with behavioural problems, such as ADHD and Autism Spectrum Disorder (ASD), as well as to more general behavioural problems [37]. In a recent critical review [11], the authors evaluated whether the data from human epidemiological studies indicate a pattern of association between ASD or ADHD and PM environmental exposure. However, this review included only one study on the association between PM and ADHD, and it found no significant associations with PM_10_. Another recent systematic review [38]studied whether air pollution caused by ambient gaseous (NO_2_, SO_2_, polychlorinated debenzo-p-dioxins and dibenzofurans, benzene) and particulate matters (PM_10_, PM_2.5_, PM_7_, polycyclic aromatic hydrocarbons, elemental carbon/black carbon) was associated with an increased risk of ADHD in children. This systematic review found seven epidemiological studies on the effects of PM and ADHD, five of which did not detect any significant association. The findings of this review indicate that there is a growing interest among epidemiologists to study the effects of air pollution exposure on neurodevelopmental disorders in children. However, there is insufficient evidence from epidemiologic studies to support a causal association between PM exposure and attention disorders. Further investigations are still needed to increase the understanding of potential health impacts from air pollution, in which the heterogeneity in terms of exposure assessment, windows of exposure, and outcome measurement should be reduced as much as possible.

### 4.1. Summary of The Evidence

The results of 9 out of 12 studies indicate an increased risk of ADHD associated with PM exposure. Although the number of studies was relatively low and the quality varied, we found that ambient PM exposure is associated with attention disorders in most of the epidemiological investigations included in this review. Also, some studies show that higher PM concentration levels tend to increase ADHD risk. However, we must take into account that the studies analyzed in this review reveal a significant heterogeneity, and the low number of epidemiological articles published on this topic does not yet allow a final verdict. Overall, we categorized only 5 of the 12 studies as high quality, whereas we grouped the other 7 as good quality. For all these reasons, the results of this systematic review should be interpreted with care, and further epidemiological studies are needed to provide conclusive evidence of a causal relationship.

### 4.2. Strengths and Limitations of the Current Review

There were several limitations to this systematic review. One was the inclusion of articles only written in English and published in peer-reviewed journals indexed in the PubMed and EMBASE databases in the review. These restrictions may have limited our search and corresponding results such that not all the relevant studies were retrieved. Furthermore, the use of certain keywords over others, and the exclusion of conference proceedings, congresses, and meeting abstracts may have also contributed to us overlooking epidemiological studies on our topic of interest. Another limitation was the heterogeneity of methodologies used in the studies, such as PM exposure assessment, and variations in outcome assessment, making it impossible to carry out a meta-analysis. We must also consider that the information on ADHD in the parents was absent. Since we know that ADHD is a highly heritable disorder, parental ADHD should be considered as a control variable. Furthermore, it is necessary to take into account that the different studies explored various windows of exposure, and this contributes to their heterogeneity. It is well known that the prenatal and postnatal phases of life represent crucial periods for brain development and that exposure in these phases can lead to a higher risk. Finally, we should also consider publication bias as another potential limitation of this review; it is known as the selective publishing of studies and it is prevalent in the scientific literature [39].

One strength of this systematic review is the extensive literature search that was carried out without a time limit. Another strength was the use of the established PRISMA guidelines to increase the clarity of reporting. Furthermore, the assessment of the quality and risk of bias can be considered additional strengths.

### 4.3. Strengths and Limitations of the Studies Included in the Review

#### 4.3.1. ADHD Assessment

Not all the studies used the same methods for the assessment of attention disorders. Some researchers collected data on attention problems using different tests, such as the Conners’ Continuous Performance Test-II (CPT-II) [40], the Stroop test [41], and the Attention Network Test (ANT) [42]. Others included studies that used the ADHD diagnosis based on DSM-IV and extracted by National Health Insurance Services. This heterogeneity in the assessment of attention disorders represents a possible source of bias and makes it difficult to compare the results of the different studies.

#### 4.3.2. Exposure Time Windows

Although the studies included in this systematic review examined the same clinical question, researchers selected different exposure time windows. The choice of different periods during which exposure is defined could have introduced an exposure misclassification [43]. The choice of different exposure time windows does not seem to be related to the conceptual framework and the biological plausibility of child-onset ADHD.

#### 4.3.3. PM Characteristics and Exposure Assessment

The assessment of ambient air pollution exposure is fundamental for determining the consequences of air pollution on health in epidemiological studies. We observed a large variability among PM exposure measurements, especially for the size of the particles investigated. PM is divided into several categories based on its size [44], and smaller particles pose a greater health risk than larger ones [45]. However, not all included studies considered the fine particulate matter (PM_2.5_) and only one assessed ultrafine particulate (PM_0.1_). We also observed heterogeneity in PM measurement methods. Some studies used land-use regression models while others obtained the data from monitoring systems.

#### 4.3.4. Confounding Variables

Confounding variables may represent an important source of bias for the under or over-estimation of the total causal effects of the exposure on the outcome [46]. Not all studies considered the same potential confounding variables and this heterogeneity could, in part, reflect the different results of the studies. For example, second-hand tobacco smoking (SHS) exposure at home was not considered as a potential confounder in some studies, although there is evidence that pre and postnatal exposure to SHS are associated with reduced neurodevelopment and intelligence and attention abilities [47]. Noise exposure is also considered a significant risk factor in neurodevelopmental disorders [48]; however, only two studies have included it as a potential confounder. Although residual confounding will likely always be present, epidemiological studies’ design should include the measurement and reporting of risk factors for which we have scientific evidence.

## 5. Conclusions

This systematic review synthesized evidence from 12 studies on the relationship between ambient particulate matter and hyperactivity and attention disorders in children. Although the majority of the included studies reported a statistically significant positive association, we observed high variability among study designs and probably high risks of bias of exposure assessment. For these reasons, together with the small number of epidemiological studies on this topic, it is not possible to establish a true causal relationship. Further high quality studies with more accurate exposure measurements are needed, as well as outcome and confounding assessment.

## Figures and Tables

**Table 1:** Description of epidemiological studies on particulate matter exposure and ADHD.

Paper	Location	Study Design	Participants	Exposure Measurement	ADHD Symptom Measured	Covariates	Results
[16]	ESCAPE (Denmark, The Netherlands, Germany, France, Italy, Spain, Sweden)	Cohort	29,127 children aged 3–10 years	land-use regression models were used for PM_10_, PM_2.5_, PMcoarse, and PM_2.5_ absorbance	A-TAC, Strengths and Difficulties Questionnaire (SDQ), CBCL, and ADHD-DSM-IV	maternal characteristics (education or socioeconomic level, country of birth, age at delivery, pre-pregnancy body mass index, height, prenatal smoking, parity), child’s sex, season at child’s birth, type of zone at child’s birth address, child’s age at assessment, and type of evaluator of the test	there was no evidence for an adverse association between air pollution exposure during pregnancy and ADHD symptoms in children.
[17]	Germany	Cohort	66,823 children aged 10–14 years	annual averages of PM_10_ were derived from freely available raster images created for Western Europe by land-use regression modeling using air pollution measurements	ADHD diagnosis was based on DSM-IV and extracted by the AOK PLUS, a German statutory health insurance company	year of birth, sex, the proportion of long-term (more than one year) and overall unemployment in home address areas, as well as healthcare access	the risk of being diagnosed with ADHD increased by a factor of 1.97 per 10 μg/m^3^ increase in PM_10_.
[18]	Japan	Cohort	33,911 children	PM < 7 μm was obtained from the environmental database managed by the National Institute for Environmental Studies in Japan	survey. Child Behavior Checklist/4–18 Japanese Edition. Three questions were related to attention problems: 1) Does your child interrupt people? 2) Can your child wait for his/her turn during play? 3) Can your child pay attention to the surrounding area when crossing the street?	sex, birth month, parity, maternal age at delivery, maternal smoking habits, maternal educational level, and paternal income during the year in which the child was born, type of municipality in which participants were born, per capita taxable income, and population density of each municipality	adjusted ORs following a one-IQR increase in SPM exposure were 1.06 (95% CI: 1.01, 1.10) for interrupting people, 1.09 (95% CI: 1.03, 1.15) for the failure to pay attention when crossing the street, 1.06 (95% CI: 1.01, 1.11) for lying, and 1.07 (95% CI: 1.02, 1.13) for causing disturbances in public.
[19]	Korea	Cohort	8396 children aged 2–10 years	Data on the ambient PM_10_ was obtained from the National Ambient Air Monitoring System. Interpolation technique using GIS tools was used to estimate the level of PM_10_ was at the unmonitored locations	ADHD diagnosis was based on DSM-IV and extracted by the National Health Insurance Service of Korea	gender, metropolitan area, and household income relative to the median, meningitis, iron deficiency anemia, and thyroid disorder	with an increase in 1 μg/m^3^ of PM_10_, the hazard ratio for childhood ADHD were 1.18 (95% CI: 1.15–1.21)
[20]	Japan	Cohort	27,527 children aged 5.5 years	PM < 7 μm was obtained from the environmental database managed by the National Institute for Environmental Studies in Japan.	survey: (1) Can your child listen without fidgeting? (2) Can your child focus on one task? (3) Does your child remain patient?	sex, birth month, maternal age at delivery, parity, maternal smoking status, maternal educational level, paternal income, municipality-level variables: residential, area, per capita income, population, density	air pollution exposure during gestation was associated with behaviours like attention
[21]	Spain	Cohort	2618 children aged 7–10 years	PM_2.5_ was measured for each pair of schools for two 1-week periods separated by 6 months. Only a pair of schools was measured each week.	computerized tests: attentional network test (ANT)	age, sex, maternal education (primary or less/secondary/university), residential neighborhood socioeconomic status, and air pollution exposure at home	high levels of traffic-related PM_2.5_ showed a slower cognitive development of schoolchildren
[22]	Boston	Cohort	267 children aged 6–7 years	PM_2.5_ was estimated by validated satellite-based spatiotemporally resolved prediction models.	children also completed the Conners Continuous Performance Test-II (CPT-II)	maternal age, race, education, prenatal/postnatal maternal smoking, parity, blood lead level at neurodevelopmental testing, child sex	for attention domains, the study did not find significant associations with prenatal PM_2.5_ exposure
[23]	Belgium	Cross-Sectional	310 children	PM_2.5_ and PM_10_ were measured at the schools with portable devices. Spatial-temporal interpolation method was used to model the daily residential exposure levels	a computer version of the Stroop Test (selective attention domain)	sex, age, education of the mother, highest rank of occupation of either parent, passive smoking, out-of-school sport activities, traffic noise, hours of computer screen time per week, and day of the	for selective attention, PM_2.5_ (*p* = 0.05) and PM_10_ (*p* = 0.02) exposures were significantly associated with the Stroop Test for an IQR increment in PM_2.5_ and PM_10_
[24]	Germany	Cohort	4745 children	annual average concentrations of PM_10_ mass, PM_2.5_ mass, and PM_2.5_ absorbance was estimated to each participant’s home add ress at birth, 10 years and 15 years using land-use regression models	hyperactivity/inattention scores were assessed using the German parent-completed (at age 10 years) and self-completed (at age 15 years) versions of the strengths and difficulties questionnaire (SDQ)	sex, age, cohort/intervention group, parental education, maternal age at birth, maternal smoking during pregnancy, child secondhand smoke exposure at age 15 years, time spent in front of a screen when child is 15 years old, time spent outside when child is 15 years old, single-parent status when child is 15 years old and parental psychopathology	significant associations were observed between hyperactivity/inattention and PM_2.5_ mass estimated to the 10 and 15 year addresses (1.12 [1.01, 1.23] and 1.11 [1.01, 1.22]) and PM_2.5_ absorbance estimated to the 10 and 15 year addresses (1.14 [1.05, 1.25] and 1.13 [1.04, 1.23])
[25]	Spain	Cohort	2715 children aged 7–10 years	ultrafine particle number (UFP; 10–700 nm) was measured simultaneously twice during 1-week periods separated by 6 months, in the warm and cold period of the year 2012	computerized tests: attentional network test (ANT)	age, sex, parent’s education and occupation, residential neighborhood socioeconomic status, air pollution exposure at home, residential and school greenness, school noise, commuting to school, smoking at home, educational quality, participation rate per school, overweight/obesity and behavioral problems	children attending schools with higher levels of UFP had a smaller improvement in cognitive development
[26]	Sweden	Cohort	3426 children aged 9–12 years	PM_10_ at residential addresses were estimated by dispersion models	A-TAC telephone interviews developed at the Institute of Neuroscience and Physiology, Gothenburg University based on the DSM-IV	parity, gender, maternal age during pregnancy, maternal smoking during pregnancy, maternal marital status at birth year, parental education, family income, and neighborhood deprivation at birth year	ambient PM_10_ level was not correlated with ADHD during pregnancy (OR = 0.85; 95% CI, 0.48–1.50) and the first year of life (OR = 0.95; 95% CI, 0.56–1.61)
[27]	India	Cross-Sectional	969 cases and 850 controls (aged 9–17 years)	PM_10_ was collected from Central and State Pollution Control Boards from their fixed-site monitoring stations	ADHD was screened following the criteria prescribed in the DSM-IV	age, sex, body mass index, socioeconomic status, parental smoking	ambient PM_10_ level was positively correlated with ADHD (OR = 2.07; 95% CI, 1.08–3.99)

Abbreviations: DSM (Diagnostic and Statistical Manual of mental disorders), BASC-2 (Behavioral Assessment System for Children, Parent Rating Scale, 2nd Edition), A-TAC (Autism-Tics, ADHD, and other Comorbidities inventory), GIS (geographic information systems), ESCAPE (European Study of Cohorts for Air Pollution Effects), EC (elemental carbon), PMcoarse (PM with aerodynamic diameters between 2.5 and 10 μm).

**Table 2:** Summary of results. Measures of association between PM exposure and ADHD expressed as Odds Ratio.

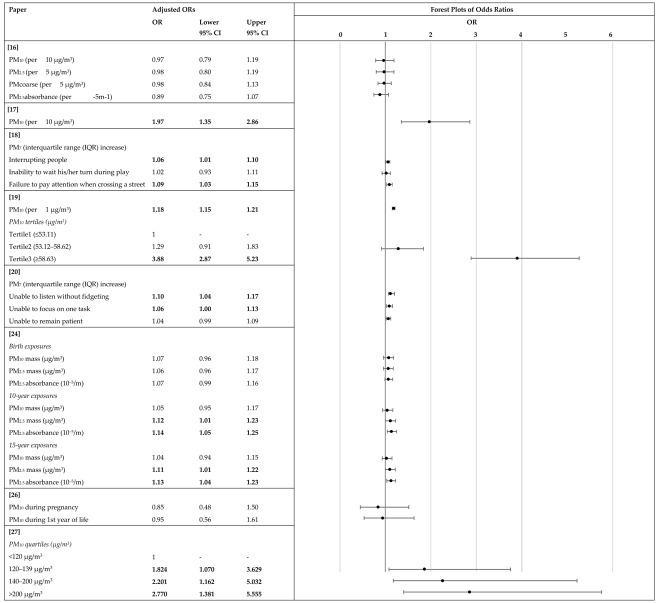

Bold values indicate that PM exposure is significantly associated with higher odds of attention disorders.

**Table 3:** Quality assessment of the included studies by the Newcastle–Ottawa scale.

Paper	Study Design	Selection	Comparability	Outcome	Total score
[16]	Cohort	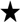 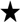 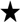 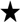	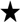 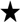	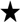	**7/9**
[17]	Cohort	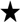 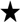 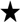 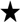	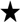 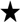	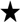 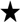	**8/9**
[18]	Cohort	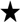 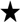 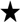 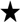	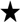 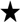	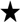	**7/9**
[19]	Cohort	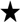 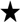 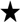 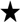	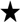	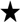 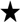	**7/9**
[20]	Cohort	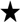 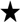 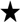	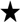 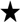	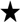 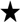	**7/9**
[21]	Cohort	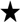 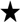 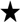 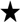	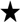 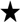	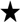 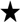	**8/9**
[22]	Cohort	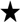 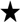 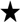	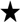 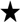	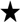 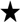 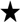	**8/9**
[23]	Cross-Sectional	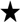 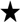 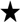 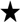	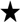 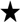	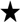 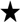	**8/9**
[24]	Cohort	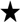 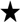 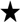 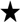	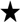 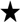	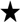	**7/9**
[25]	Cohort	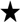 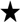 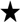	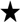 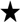	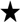 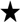	**7/9**
[26]	Cohort	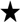 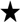 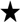	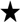 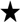	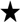 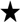	**7/9**
[27]	Cross-Sectional	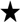 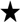 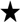 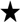	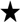 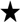	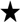 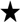	**8/9**

**Table 4:** Heat map of the risk of bias rating for 12 studies.

	Key criteria			Other Criteria				
Paper	Exposure Assessment	Outcome Assessment	Cofounding Bias	Selection Bias	Attrition/ Exclusion Bias	Selective Reporting Bias	Conflict of Interest	Other Sources of Bias
[18]								
[17]								
[19]								
[20]								
[21]								
[16]								
[22]								
[25]								
[23]								
[28]								
[24]								
[26]								
								
**Risk of Bias**	**Low**		**Probably low**		**Probably high**		**High**	

## Supplementary Materials

The following are available online at https://www.mdpi.com/1660-4601/17/1/67/s1. File S1: PRISMA checklist. File S2: PECOS criteria for inclusion and exclusion of studies. File S3: Quality and risk of bias assessment for each study.
