# Supplementary material for: Particulate Matter Exposure and Attention-Deficit/Hyperactivity Disorder in Children: A Systematic Review of Epidemiological Studies
Source: Int J Environ Res Public Health. 2019 Dec 20;17(1):67. doi: 10.3390/ijerph17010067 (PMC6982101; doi:10.3390/ijerph17010067)
Supplement: Supplementary file 1 [file ijerph-17-00067-s001.zip › file.2.pdf]

Particulate matter exposure and Attention-Deficit/Hyperactivity Disorder in children: A systematic review of epidemiological studies.

Supplementary B. PECOS criteria for inclusion and exclusion of studies

Detailed PECOS (population, exposure, comparison, outcome, study type) table for the systematic review on particulate matter and ADHD.

	Population	Exposure	Comparison	Outcome	Type of Study
**Included**	Child population	PM air pollution exposure	Not applicable	ADHD	Observational studies (cohort, case-control and cross sectional)
**Excluded**	Adults, in vitro and animal studies	Other air pollutants (e.g. ozone, NO_X)	Not applicable	Other neurodevelopmental disorder (e.g. autism)	Controlled trials Letters to the editor Reviews<br>Case report<br>Interventional studies<br>Abstracts
