# Supplementary material for: Particulate Matter Exposure and Attention-Deficit/Hyperactivity Disorder in Children: A Systematic Review of Epidemiological Studies
Source: Int J Environ Res Public Health. 2019 Dec 20;17(1):67. doi: 10.3390/ijerph17010067 (PMC6982101; doi:10.3390/ijerph17010067)
Supplement: Supplementary file 1 [file ijerph-17-00067-s001.zip › file.3.pdf]

Particulate matter exposure and Attention-Deficit/Hyperactivity Disorder in children: A systematic review of epidemiological studies.

Supplementary C. Quality and risk of bias assessment for each study

The Newcastle-Ottawa scale (Wells G 2013) was adopted in this review to evaluate the quality of cohort and cross-sectional study respectively.

The Office of Health Assessment and Translation (OHAT) by the National Institutes of Environmental Health Sciences National Toxicology Program (NEHS-NTP) (OHAT 2015) and Navigation Guide by the University of California (Lamet al. 2016; Woodruff and Sutton 2014) was adopted to evaluate risk of bias for each included study.

References

- Wells G, S.B., O'Connell D, Peterson J, Welch V, Losos M, Tugwell P. The Newcastle-Ottawa Scale (NOS) for assessing the quality of nonrandomised studies in meta-analyses. http://www.ohri.ca/programs/clinical_epidemiology/oxford.asp; 2013
- Zhao, T., Markevych, I., Romanos, M., Nowak, D., & Heinrich, J. (2018). Ambient ozone exposure and mental health: Asystematic review of epidemiological studies. Environmental research.
- OHAT. Handbook for Conducting Systematic Reviews. Office of Health Assessment and Translation (OHAT) Division of the National Toxicology Program National Institute of Environmental Health Sciences; 2015
- Woodruff, T.J.; Sutton, P. The Navigation Guide systematic review methodology: a rigorous and transparent method for translating environmental health science into better health outcomes. Environ Health Perspect 2014;122:1007-1014

**1. Forns et al., 2018**		
**Design**	**Cohort study**	
Participants	Human, aged 3-10 years	
Exposure	PM10, PM2.5, PMcoarse and PM2.5 absorbance.	
Comparison	29127 children followed up from 1992 through 2008	
Outcomes	Attention-Deficit/Hyperactivity Disorder	
**Quality Assessment**		
**Newcastle-Ottawa Quality Assessment Scale-Case Cohort Study**		**Author's judgement**
**Selection**	Representative of the exposed cohort	*
	Selection of the non-exposed cohort	*
	Ascertainment of exposure	*
	Demonstration that outcome of interest was not present at start of study	*
**Comparability**	Comparability of cohorts on the basis of the design of analysis	**
**Outcome**	Assessment of outcome	
	Was follow-up long enough for outcome to occur	*
	Adequate of follow up of cohorts	
**Risk of Bias Assessment**		
	**Bias Domain**	**Author's judgement**
**Key criteria**	Detection bias, exposure assessment	Probably high
	Detection bias, outcome assessment	Probably high
	Confounding bias	Low risk
**Other criteria**	Selection bias	Probably low
	Attrition/exclusion bias	Low risk
	Selective reporting bias	Low risk
	Conflict of interest	Low risk
	Other sources of bias	Probably low

2. Markevych et al., 2018		
**Design**	**Cohort study**	
Participants	Human, aged 10-14	
Exposure	PM_{10}	
Comparison	66,823 children followed up from 2000 through 2014	
Outcomes	Attention-Deficit/Hyperactivity Disorder	
**Quality Assessment**		
**Newcastle-Ottawa Quality Assessment Scale-Case Control Study**		**Author's judgement**
**Selection**	Representative of the exposed cohort	*
	Selection of the non-exposed cohort	*
	Ascertainment of exposure	*
	Demonstration that outcome of interest was not present at start of study	*
**Comparability**	Comparability of cohorts on the basis of the design of analysis	**
**Outcome**	Assessment of outcome	*
	Was follow-up long enough for outcome to occur	*
	Adequate of follow up of cohorts	
**Risk of Bias Assessment**		
	**Bias Domain**	**Author's judgement**
**Key criteria**	Detection bias, exposure assessment	Probably high
	Detection bias, outcome assessment	Low
	Confounding bias	Probably low
**Other criteria**	Selection bias	Probably low
	Attrition/exclusion bias	Probably low
	Selective reporting bias	Low
	Conflict of interest	Low
	Other sources of bias	Probably low
